# RASSF1A, puppeteer of cellular homeostasis, fights tumorigenesis, and metastasis—an updated review

**DOI:** 10.1038/s41419-019-2169-x

**Published:** 2019-12-05

**Authors:** Fatéméh Dubois, Emmanuel Bergot, Gérard Zalcman, Guénaëlle Levallet

**Affiliations:** 1 Normandie University, UNICAEN, CEA, CNRS, ISTCT/CERVOxy group, GIP CYCERON, Caen, France; 2 Department of Pathology, CHU de Caen, Caen, France; 3 Department of Pulmonology & Thoracic Oncology, CHU de Caen, Caen, France; 4 U830 INSERM “Genetics and biology of cancers, A.R.T group”, Curie Institute, Paris, France; 5 Department of Thoracic Oncology & CIC1425, Hôpital Bichat-Claude Bernard, Assistance Publique Hôpitaux de Paris, Université Paris-Diderot, Paris, France

**Keywords:** Tumour-suppressor proteins, Biomarkers, Oncogenesis

## Abstract

The Ras association domain family protein1 isoform A (RASSF1A) is a well-known tumor-suppressor protein frequently inactivated in various human cancers. Consistent with its function as a molecular scaffold protein, referred to in many studies, RASSF1A prevents initiation of tumorigenesis, growth, and dissemination through different biological functions, including cell cycle arrest, migration/metastasis inhibition, microtubular stabilization, and apoptosis promotion. As a regulator of key cancer pathways, namely Ras/Rho GTPases and Hippo signaling without ignoring strong interaction with microtubules, RASSF1A is indeed one of the guardians of cell homeostasis. To date, as we approach the two decade anniversary of RASSF1A’s discovery, this review will summarize our current knowledge on the RASSF1A key interactions as a tumor suppressor and discuss their impact on cell fate during carcinogenesis. This could facilitate a deeper understanding of tumor development and provide us with new strategies in cancer treatment by targeting the RASSF1A pathway.

## Facts

- RASSF1A is one of the prototypical tumor-suppressor gene universally inactivated in human malignancies.
- RASSF1A is a prognostic biomarker and predicts chemosensitivity in cancer.
- The scaffold activity of RASSF1A enables its action as a nexus for the coordination of numerous signaling pathways that control cell fate, cell metabolism, cell communication cell motility, cell growth and division, and cell death.
- As a tumor-suppressor gene, RASSF1A mainly acts as a crossroad of three intertwined molecular signaling mechanisms including Ras/Rho GTPases, Microtubules, and Hippo pathway.

## Open questions

- Interest of the restriction of intercellular communication via tunneling nanotubes (TNTs) by RASSF1A.
- Control of cell metabolism by RASSF1A under hypoxia.
- Influence of the tumor microenvironment on the functionality of RASSF1A.

## Clinical implications of RASSF1A inactivation

### Universal silencing of RASSF1A in human cancers

Described almost two decades ago as a Ras-GTP binding protein, RASSF1A is one of the prototypical tumor-suppressor genes frequently inactivated in >40 types of human malignancies, including lung, breast, prostate, glioma, neuroblastoma, multiple myeloma, and kidney cancer^1–3^. Although promoter hypermethylation and loss of heterozygosity of the remaining allele are the most common molecular mechanism of silencing the *RASSF1* gene, RASSF1A can also be inactivated by protein degradation or point mutation^4^. MicroRNAs, including miR-602, miR-181a/b, and miR-214-3p, can also downregulate RASSF1A in several cancers^5–7^.

### Diagnostic and prognostic interests of RASSF1A inactivation in cancers

The research for RASSF1A inactivation has been steadily gaining prominence due to both diagnostic and prognostic interests in cancer development^3^. RASSF1A hypermethylation being a key early event during carcinogenesis, the detection of a methylated *RASSF1* promoter in plasma circulating tumor DNA is an attractive biomarker for early detection of various cancers^8,9^. Methylation of the *RASSF1* promoter is rarely found in normal tissues, while it is correlated with high-grade tumors and is predictive of poor prognosis and more aggressive clinical phenotypes in patients^10–12^. Besides, the restoration of RASSF1A expression by demethylating agents suppresses tumor cell growth^13^.

### Interest of RASSF1A inactivation in patients’ responsiveness to chemotherapy

RASSF1A methylation assessed in tumors or blood is also predictive for patients’ responsiveness to neoadjuvant chemotherapy^11,14^. Indeed, the phase III trial investigation by French Intergroup (IFCT) showed the predictive values of RASSF1A methylation pattern, for predicting survival following neoadjuvant chemotherapy in patients with stage I–II NSCLC: a poor median overall survival was observed in patients with methylated *RASSF1* promoter treated with gemcitabine (30.3 months) compared with those treated with paclitaxel (70 months)^11^.

To understand how RASSF1A silencing applies to cancer, we will review the RASSF1A structure and principal interacting partners, and elaborate on how RASSF1A inactivation can be placed in the context of distortions of larger signaling networks that fuel initiation and progression of cancer. Overall, as RASSF1A methylation represents a strong potential for clinical utility, increasing our knowledge of its interaction and subsequent activities is key to identifying new therapeutic paths.

## RASSF1A structural features and principal interacting partners

### RASSF1A structural features

**Fig. 1:**
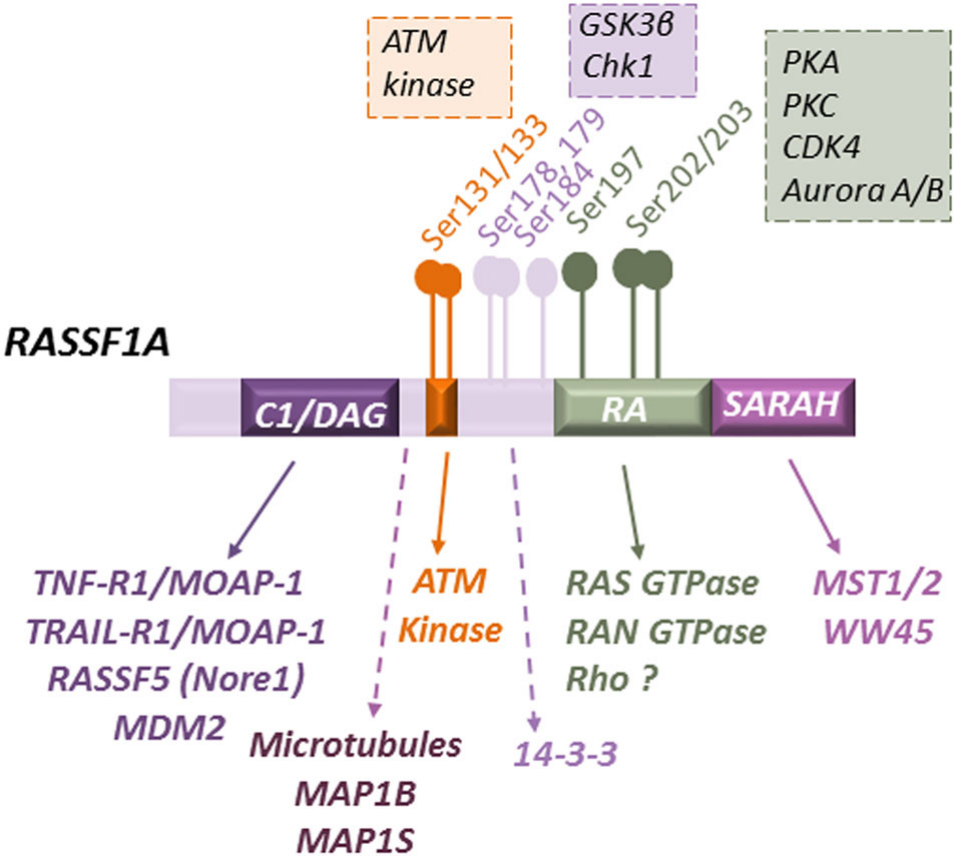
The molecular structure of RASSF1A and its interacting molecules. The cytosolic RASSF1A protein, encoded by the RASSF1 gene, is a scaffolding protein without any enzymatic activity. Multiple interaction motifs located within RASSF1A enable recruitment of specific proteins and regulation of downstream signaling pathways^4^. In addition, multiple phosphorylation sites exist through the RASSF1A molecule, which are targeted by a diverse array of kinases (listed in boxes) and influences its activity^28–34^.

As a member of the RASSF family, RASSF1A is a best-characterized isoform of the *RASSF1* gene located on the chromosome 3p21.3, a genomic region with high density of tumor-suppressor genes susceptible to epigenetic silencing and/or deletion in numerous cancers (Fig. 1)^15^. Expressed in normal human tissues, RASSF1A exerts its functions through its scaffold properties at the crossroads of many intracellular signaling to coordinate, integrate, and facilitate efficient cell signaling, through direct or indirect interactions with multiple structural and signaling proteins^4^.

The N-terminus of RASSF1A harbors a cysteine-rich domain (CRD), similar to the diacylglycerol (DAG)/Phorbol ester-binding domain of the protein kinase C family (C1/DAG domain), which is involved in the associations of RASSF1A with the death receptors complex (TNF-R1/MOAP-1 or TRAIL-R1/MOAP-1)^16^. In addition, the N-terminal portion of RASSF1A is responsible for homo- and heterodimerization with RASSF5 (Nore1), another member of the RASSF family^17^. Furthermore, RASSF1A holds a consensus site for ATM phosphorylation on serine 131, called ataxia telangiectasia mutant (ATM) domain^18^. Two single-nucleotide polymorphisms located in this domain have already been identified in some human tumors^19^.

The Ras/Rap-associated (RA) domain, the main structural feature of the RASSF family^20^, allows for a specific interaction with activated members of the Ras family. However, the RA domain of RASSF1 displays rather weak affinity^17,21,22^, in contrast to RASSF5, which interacts with several Ras-like GTPases, through much greater affinity^23,24^. Most likely, the heterodimerization of RASSF1A with RASSF5 can indirectly connects the Ras signaling pathway with the RASSF1A protein^17^.

The C-terminus of RASSF1A contains a Salvador/RASSF/Hpo (SARAH) domain, a coiled–coil structure only found in two other proteins: the regulatory protein WW45 (human homologue of the Drosophila protein Salvador) and the serine/threonine kinases MST1 and MST2 (human homologues of the Drosophila kinase Hippo/hpo)^25^. The SARAH domain mediates the direct interactions between RASSF1A and these proteins, the core members of the Hippo signaling pathway^26^.

RASSF1A also contains a region between amino acids 120 and 185 necessary for the association with the microtubules (MTs), major partners of RASSF1A^27^, and a PXXP-like sequence that allows its association with SH3-containing proteins. However, no function has been assigned to the potential SH3 binding PxxP motif on RASSF1A^21^. Finally, the three serines at position 175, 178, and 179 of RASSF1A are a potential docking site for an endogenous 14–3–3 scaffold protein, which maintains RASSF1A inactive in the cytoplasm^28^.

It is of note that multiple phosphorylation sites exist throughout the RASSF1A molecule, targeted by a diverse kinases, which contribute to the complexity of the RASSF1A interactome by providing additional docking sites for other structural and signaling components. These include PKA^29^, PKC^30^, CDK4^31^, Aurora A/B kinases^32,33^, Chk1 kinase^34^, as well as GSK3β (glycogen synthase kinase) kinase^28^ and ATM kinase^18^.

### RASSF1A and microtubules (MTs)

**Fig. 2:**
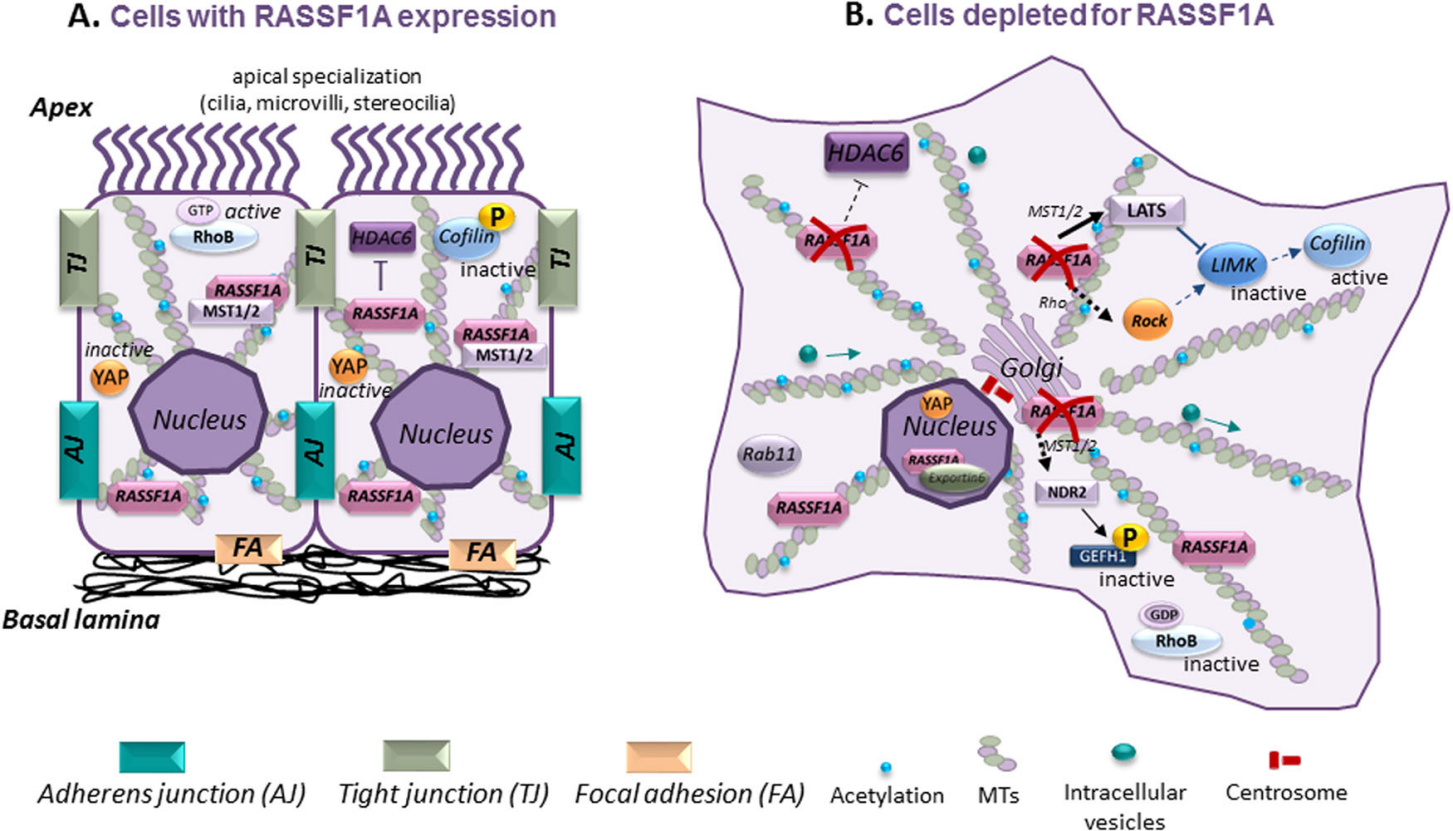
RASSF1A as a master-organizer of epithelial cell differentiation maintenance and cell trafficking. RASSF1A influences the cytoskeleton directly (through HDAC6 inhibition^37^) or via Rho GTPases or/and LIMK/Cofilin signaling to promote YAP cytoplasmic accumulation in inactive form^56^. Furthermore, RASSF1A enhances stabilization of focal adhesion and cell–cell junctions^56^. RASSF1A stabilizes the microtubules through its inhibitory action on HDAC6 deacetylase^37^ to build a long, reliable tracks for MT-dependent transport^85^. Furthermore, RASSF1A-associated MTs acetylation is necessary for the centrosome and Golgi organization^39–41,93^. RASSF1A is also able to influence LIMK/Cofilin activity^56^ and Rab GTPase signaling^86^, which play crucial roles in intracellular transport and exosome release^87,88^.

This association is at the heart of RASSF1A functions as a tumor-suppressor protein. Indeed, in the absence of this property, RASSF1A fails to control cell proliferation and apoptosis^27,35^. RASSF1A’s interaction with the MTs causes stable circular or bundled perinuclear rings instead of polarized filaments with plus (growth) and minus (shrinkage) ends^36^. Mechanistically, RASSF1A interacts and inhibits the deacetylation function of HDAC6 (histone deacetylase 6), resulting in an increase of acetylated MTs, which are more stable and long-lived, but less dynamic^37^ (Fig. 2).

RASSF1A’s interaction with the MTs is also regulated by phosphorylation: in a poorly phosphorylated state, RASSF1A stably associates with microtubules, however, an increase in the protein’s phosphorylation, by the kinases PKA, PKC, Chk1, and Aurora A, decreases its affinity for MTs, causing their disorganization^30,32^.

Moreover, an analysis for RASSF1-interacting proteins showed that 70% of interacting peptides were homologous to microtubule-associated proteins (MAPs)^36,38^. Therefore, RASSF1A can also interact with MTs indirectly through association with MAPs, such as MAP1B, MAP4, and C19ORF5 (chromosome 19 open-reading frames 5)^4^. While association with either MAP1B or C19ORF5 increases the MTs growth and stability, the interaction with MAP4 impedes both the depolymerization rate and catastrophe frequency^4^.

Of importance, RASSF1A also localizes at the centrosome, which serves as a major microtubule-organizing center (MTOC)^39,40^. RASSF1A overexpression inhibits centrosome separation^39^, whereas knockdown of RASSF1A causes multiple centrosome formations^41^. Interestingly, multiple RASSF1A-binding proteins also localize to the centrosome, including the members of the Hippo pathway MST/WW45/LATS, NDR complex^25,42^, C19ORF5^43^, Aurora-A^44^, and γ-tubulin^45^, suggesting that RASSF1A may either recruit these proteins to the MTOC or vice versa. These data further link RASSF1A to the MTs network.

Finally, RASSF1A has not been demonstrated to co-localize to actin or intermediate filaments. However, given the coordination of the organization of MTs and actin filaments in cells and the alteration of the actin cytoskeleton induced by RASSF1A depletion^46^, the impact of RASSF1A on actin seems to require intermediates. For instance, the LATS kinases regulate the activity of proteins involved in actin filament nucleation and elongation, such as LIMK or Zyxin^47^ (Fig. 2). In addition, RASSF1A interacts with MAP proteins, identified as key players that directly cross-link the two cytoskeletons^48^. As a functional consequence, RASSF1A probably coordinates polarized cell migration and cell trafficking, which are the prime instances, in which actin and microtubules become physically linked. Overall, RASSF1A’s tumor-suppressor function could at least partly be depending on its modified interaction with the MT/cytoskeleton network.

### RASSF1A and Hippo pathway members, inseparable partners

RASSF1A is an upstream component of the Hippo pathway, a master regulator of cell survival, proliferation, mechano-transduction, and organ size during development^49^. This pathway is regulated at different levels by a myriad of intrinsic and extrinsic signals, but canonical signaling involves a kinase cascade (namely MST1/2, LATS1/2, NDR1/2 in mammals) that once activated, phosphorylates and inhibits the downstream final effectors YAP and TAZ^50,51^. When the core kinases are inactive, YAP/TAZ are unphosphorylated and translocate into the nucleus to interact with various transcription factors such as TEAD1-4, p73, RUNX, or SMAD^52^. The activity of the Hippo kinases is supported by two adaptor proteins, the WW-domain containing scaffold protein Salvador (SAV1 or WW45) and the Mps One Binder 1 (MOB1), which bind to and favor MST1/2 and LATS1/2 phosphorylation, respectively, leading to YAP/TAZ phosphorylation and inhibition^51^.

As previously mentioned, RASSF1A binds MST1/2 kinases and adaptor protein WW45 (SAV1) directly via the SARAH motif^51^. This interaction allows RASSF1A to regulate apoptosis in response to DNA damage or replication stress^4,53^, autophagy initiation^54^, epithelial–mesenchymal transition (EMT), invasive phenotype, and elevation in tissue stiffness^55–57^, roles that we will describe later in this review.

### RASSF1A and superfamily of Ras small GTPases

RASSF1A binds with low affinity and only to the farnesylated form of the Ras proteins and most preferentially to K-Ras^4,13,58^. RASSF1A functions primarily as the main Ras death effector, interaction of K-Ras with RASSF1A either activates the MST2-LATS1 apoptotic pathway^58,59^, or enhances the interaction of RASSF1A and MOAP-1, further promotes RASSF1A’s ability to induce Bax translocation to the mitochondria and cell death^60,61^. The K-Ras/RASSF1A association can also enhance MDM2 degradation by the proteasome, in turn causing enhanced p53 stability^31^. The stabilization of the MTs by RASSF1A is enhanced by activated K-Ras, and so RASSF1A connects Ras to the control of MTs dynamics^45^. RASSF1A interaction with Ran GTPase as well as Rap1A also controls MTs behavior^22,62^.

Nevertheless, K-RAS and RASSF1A seem to have a more intricate connection than simple upstream/downstream mediators. Indeed, both RAF/MAPK and PI3K/AKT pathways, the two best-characterized Ras mitogenic effectors, are modulated by RASSF1A^63,64^. For example, as MST2 binding to RAF-1 serves to suppress RAF-1 activation, RASSF1A modulates the RAF-1 activity due to competition with MST2 for RAF-1 binding^65,66^. The second example is the suppression of AKT anti-apoptotic activity by the RASSF1A/Hippo pathway^67,68^. A recent report showed a clear upregulation of PI3K/AKT and RAL activities in the tumors with suppressed RASSF1A^69^.

Ras signaling also stimulates several pathways and signals toward the Rho GTPases family (RhoA/B/C, Rac, CDC42), which are well-known master regulators of cell adhesion and motility^70^. RASSF1A contributes in the regulation of the Rho family, and therefore to the coordination of their downstream signaling components. RASSF1A depletion is notably associated with upregulation of Rac1 activity^46^, and direct interaction of RASSF1A with RhoA causes the suppression of RhoA transforming activity^71^. RASSF1A also modulates the activity of RhoB GTPase, which functions as RhoA/RhoC antagonists^72^, through fine-tuning GEF-H1 activity by inducing its phosphorylation via NDR2 kinase^56,73^. More recently, Rheb, a Ras-related small GTPase, was shown to form a complex with RASSF1A to coordinate Hippo and TOR signaling^74^. Ultimately, the opposing function of RASSF1A seems to play a critical and cooperative role in determining the fate of the Ras GTPases family signaling as a proto-oncogene during carcinogenesis^64,75^.

## The role of RASSF1A’s scaffold activity in prevention of carcinogenesis

As a scaffolding protein, RASSF1A contributes to the recruitment of specific kinases and phosphatases, oncoproteins, and structural proteins, involved in intracellular signaling cascades. We focus here on the role of RASSF1A at the crossroads of three intertwined molecular signaling mechanisms, including Ras/Rho GTPases, MTs, and the Hippo pathway.

### RASSF1A and protection of epithelial phenotype

Abnormal activation of EMT is related to invasion and metastasis of tumor cells to adjacent tissues, which is associated with decreased therapeutic effectiveness and the vast majority of cancer-related deaths^76^ (Fig. 2). Microarray expression profiling in A549 cancer cells provided our first glimpse of RASSF1A’s role in controlling cell migration by demonstrating the significant upregulation of the genes involved in cell adhesion and motility after RASSF1A expression^77^. Consistently, the increase of MTs’ stability through RASSF1A’s control of HDAC-6 activity is another factor responsible for RASSF1A’s implication in control of cell motility and invasion^2,36,37^. In addition, RASSF1A-depleted cells displayed increased cell migration and diminished cell–cell adhesion, in a PI3K- and Rac1-dependent manner^46^. These data are further supported by reports showing increased invasiveness and metastasis of RASSF1-methylated tumors^55^. Consistently, loss of RASSF1A increased cell motility and invasion capacities favoring tumor grafting of bronchial cancer cells and their metastatic dissemination in SCID mice^56^. Mechanistically, RASSF1A depletion enhances destabilization of adherent junctions, which further stimulates the conversion of epithelial cells to the more malignant mesenchymal phenotype. Low RASSF1A expression also increases cofilin activity^56^, which consequently promotes cell mobility during tumor migration and invasion^78^.

Another major consequence of RASSF1A depletion is nuclear accumulation of the Hippo pathway transcriptional co-activator YAP^55,73^, which is also an established regulator of EMT^52^. Mechanistically, methylation of RASSF1A promoter leads to RASSF1C transcription by the alternative use of the second *RASSF1* promoter. RASSF1C, unlike RASSF1A, has oncogenic effects^79^. The opposite action of RASSF1C has been recently reviewed by our group^80^. Along the same vein, TGF-β, one of the principal EMT inducers^81^, targets the degradation of RASSF1A to facilitate YAP/SMAD2 nuclear translocation^82^. Interestingly, nuclear YAP regulates TGF-β-induced transcriptional programs, resulting in increased cell migration and invasion^83^. Of importance, YAP is not only downstream of EMT but also an active inducer of EMT through regulating multiple EMT-related genes^52^. Adding to the complexity, nuclear translocation of YAP in RASSF1A-depleted cells also depends on inactivation of the GEF-H1 (GTPase exchange factor H1), and subsequent inactivation of the RhoB GTPase^56,73^.

In addition, modulating MTs’ dynamics affects the activation of Rho GTPases and consequently the formation of the lamellipodia, filopodia, and cell migration. Therefore, loss of RASSF1A influences the cytoskeleton either directly or via Rho GTPases and/or LIMK/Cofilin signaling to promote YAP nuclear localization during the acquisition of invasive hallmarks. Collectively, these actions suggest that RASSF1A inactivation is not only a prognostic biomarker of primary tumors but also predict a higher potential for tumor cells metastasis.

### RASSF1A, cell trafficking and communication

If RASSF1A modulates stable/acetylated versus dynamic MTs^37,84^, then one might expect RASSF1A to correlate with the configuration of long, reliable tracks for MT-dependent transport^85^. This hypothesis is concordant with previous reports showing the ability of RASSF1A to influence LIMK/Cofilin activity^56^ and Rab GTPase signaling^86^, which play crucial roles in intracellular transport and exosome release^87,88^ (Fig. 2). Moreover, another study suggests the link between RASSF1A and endosomal trafficking through interaction with tumor necrosis factor receptor 1, and states that TNF-R1 internalization is probably dependent on a stable microtubular network influenced by RASSF1A^16^. In addition, a recent report uncovered a new role for endogenous RASSF1A as a regulator of actin nucleocytoplasmic trafficking by corroborating binding of transport receptor exportin-6 to RAN GTPase^89^. Thereby, given the ability of dysregulated vesicle trafficking to promote cancer cell invasion and metastasis^90^, and exosomes to create a pre-metastatic niche^91,92^, their malfunction could contribute to cancer progression of RASSF1A-depleted cells.

Moreover, RASSF1A-associated MTs’ acetylation is necessary for proper Golgi complex integrity and organization. Consistently, loss of RASSF1A results in significant Golgi fragmentation in both normal and cancerous cells, and disturbs proper cell polarity and migration^93^. Reorientation of a cohesive Golgi apparatus to a position ahead of the nucleus in the direction of migration facilitates the efficient delivery of essential proteins, such as metalloproteinases to the leading edge^94^. Consequently, disrupted Golgi organization is also indicative of a polarized trafficking/secretion defect, leading to cancer progression and metastasis^95^. RASSF1A may also be acting to coordinate Golgi position independent of MTs or acetylation, through modulating the activities of other proteins known to influence Golgi’s structure/function, including Aurora A and/or MAP1B^96,97^.

**Fig. 3:**
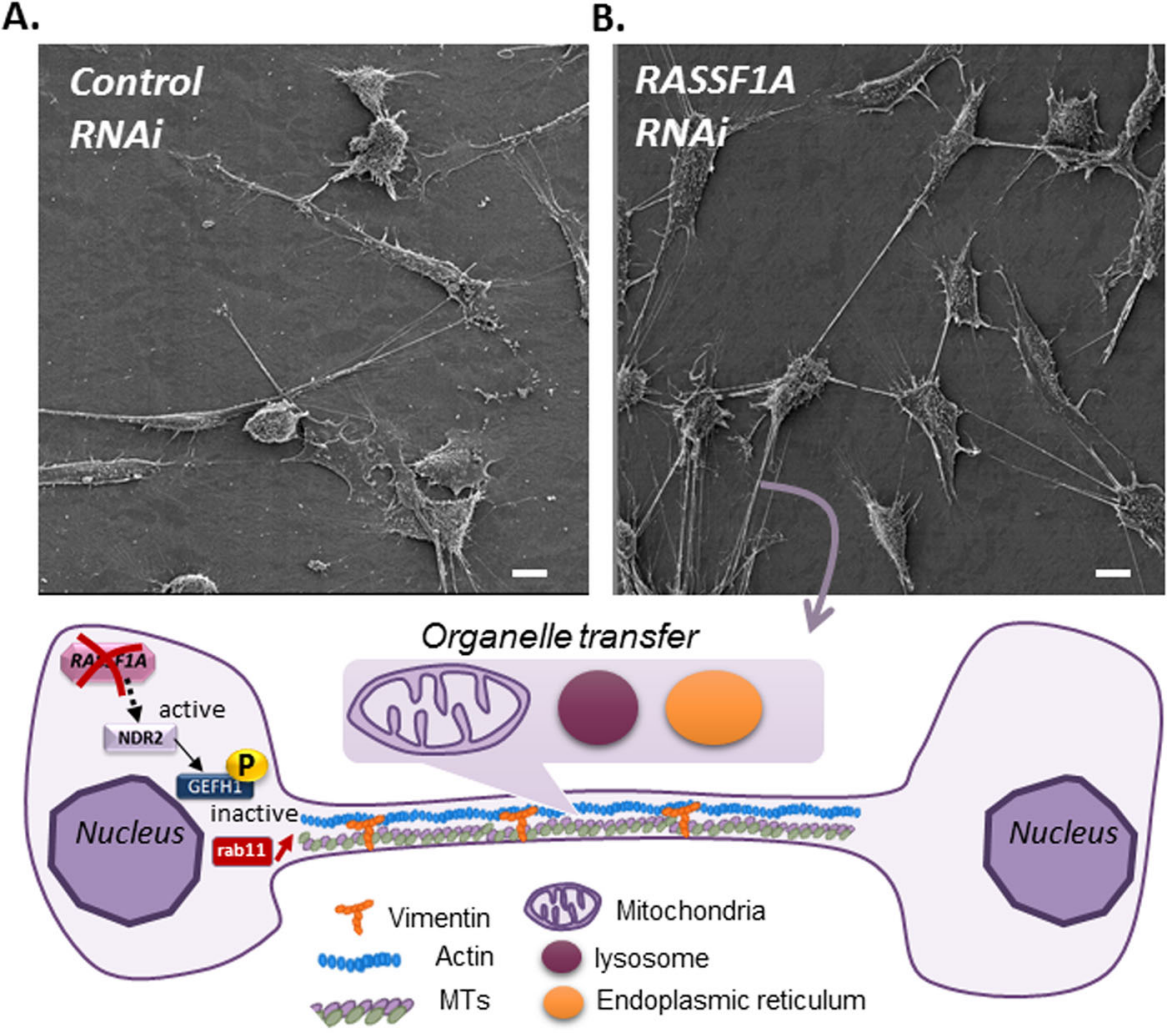
RASSF1A depletion enhances TNTs formation. H2452 cells transfected transiently with (**a**) control or (**b**) RASSF1A RNAi were fixed and subjected to scanning electronic microscopy. For further information please see ref. ^86^. Scale bar = 10 µm.

Surprisingly, we have identified a novel role for RASSF1A in regulating the formation of long membrane protrusions known as tunneling nanotubes (TNTs) ^86^, forming a bridge between cells far from each other^98^. By facilitating intercellular communication between cells, TNTs play a critical role in cancer^99,100^. Depletion of RASSF1A increases both TNTs’ formation and TNT-mediated intercellular propagation of different organelles such as mitochondria or lysosome in a bronchial or mesothelioma cell lines (Fig. 3). Mechanistically, RASSF1A depletion induces GEFH-1 inactivation, leading to Rab11 accumulation and subsequent exosome release, which in turn contributes to TNTs’ formation^86^ (Fig. 3). Moreover, the formation of TNTs is accompanied by cytoskeleton remodeling and is stimulated through disruption of cell–cell junctions upon EMT^55,101^, offering further mechanistic insights into how RASSF1A might control TNTs formation^56,86^. Thus, as we strive to understand the impact of RASSF1A depletion across a variety of cancers, it is important to take into account the beneficial or deleterious impact of long distance communications, either by exosomes or by TNTs, in cancer initiation, progression, and metastasis. Such functions could pave the way for new strategies for cancer therapy.

### RASSF1A and cell cycle regulation

**Fig. 4:**
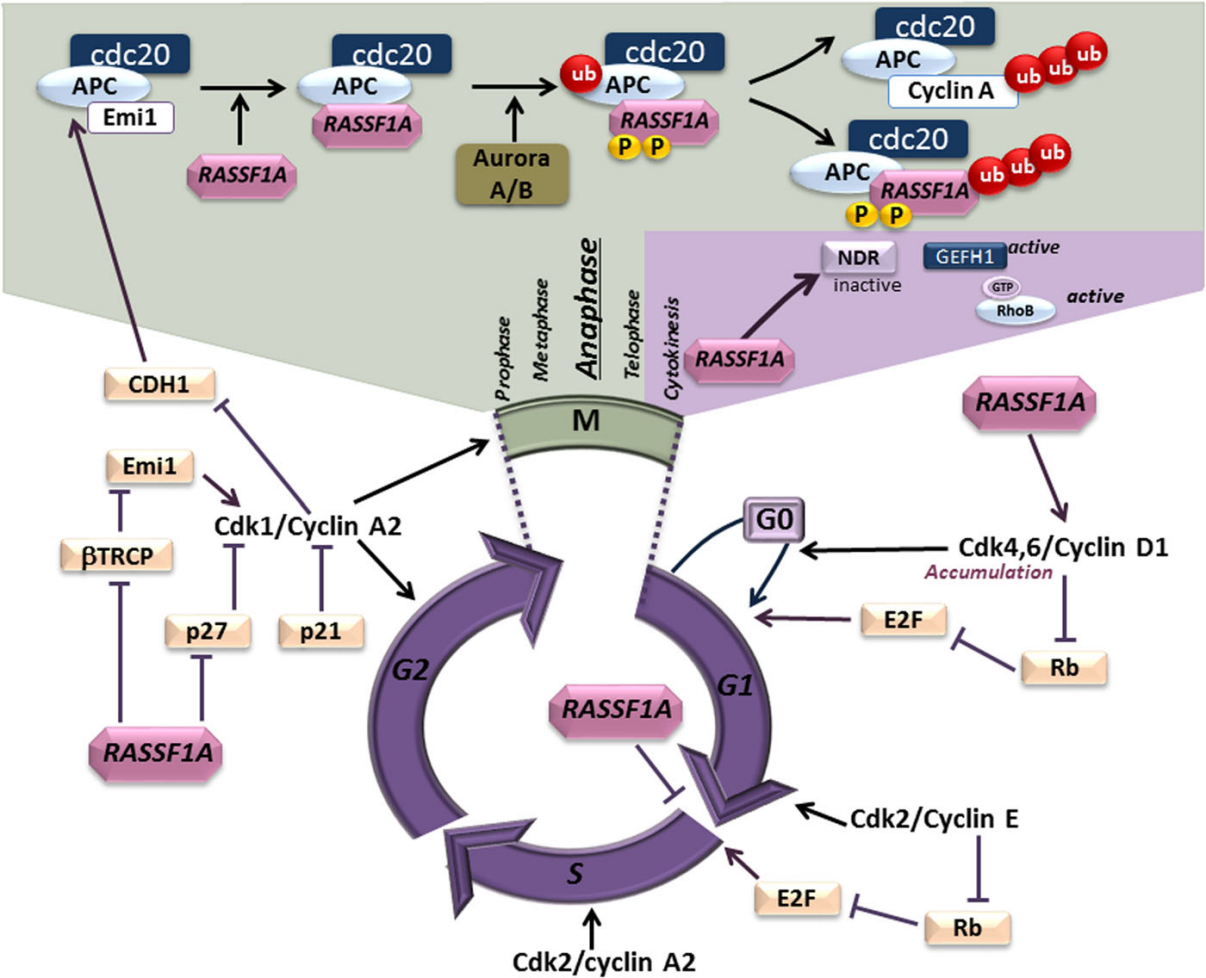
RASSF1A control cell proliferation and cell cycle progression. Through its scaffolding properties, RASSF1A regulates a subset of proteins involved at different stages of cell cycle^4,64^. Accordingly, the tumor-suppressor activity of RASSF1A has been reported to reside mainly in its ability to restrict cell cycle progression^35^.

RASSF1A expression and protein levels fluctuate during the cell cycle^32,102^, and RASSF1A’s localization is dynamic and varies according to the different stages, from the centrosome in prophase to the spindle poles in metaphase and anaphase, and to the midbody during telophase^40,43,75^. Consequently, through scaffolding activity, RASSF1A regulates a subset of proteins involved in controlling cell cycle progression (Fig. 4). For instance, RASSF1A inhibits cell cycle progression at the G1-S transition by preventing the accumulation of Cyclin D1^19^ through the decrease of JNK kinase activity^103^, and by repressing Cyclin A2 synthesis through promoting the interaction of transcription factor p120^E4F^ on its promoter^104^. During G2, RASSF1A causes cell cycle arrest, by repressing the p27 and β-TRCP proteins, which leads to cyclin A2 accumulation^40,105^. Subsequently, the degradation of RASSF1A by the SCF E3 and/or CUL4A E3 ubiquitin ligase complex allows cell progression through mitosis^31,102^.

During prometaphase, the interaction of RASSF1A with MAP1S (C19ORF5) confines RASSF1A at the mitotic pole and centrosome^32,41^, allowing RASSF1A to block mitotic progression at prometaphase through interaction and inhibition of the APC/Cdc20 complex^41^. Once again at a later stage, RASSF1A’s phosphorylation by the mitotic kinase Aurora-A, allows the progression of cell division toward the anaphase by causing degradation of RASSF1A through ubiquitination^32,33,106^. However, during cytokinesis, the phosphorylation of RASSF1A by Aurora-B stimulates the recruitment of Syntaxin16, a member of the t-SNARE membrane fusion protein family, to the midbody to ensure completion of cytokinesis^33^.

Consequently, RASSF1A depletion moderately accelerates mitosis through premature degradation of mitotic cyclins^41^, but mainly causes an increased number of cytokinesis abnormalities including binucleate and interconnected cells^25,43,73^. One mechanistic explanation could be attributed to overexpression of Anillin and/or to a lack of Citron kinase after RASSF1A knockdown through the RASSF1A/NDR2/GEF-H1/RhoB axis^73^. However, it is not clear whether this defect results from a direct function of RASSF1A or is an indirect consequence of disrupting the Rho GTPases activity^56,71^, and/or MT dynamics. Indeed, RASSF1A’s localization and/or interaction with the MTs is essential for faithful segregation of genetic material^10,45^. Accordingly, loss of MT association abolishes the ability of RASSF1A to modulate cell cycle progression^25,34,41^. As discussed above, another explanation may come from a defect in the vesicle–membrane trafficking, which is required for proper cell abscission^107^.

During the cell cycle, RASSF1A also contributes significantly to the DNA repair process itself through two different pathways. By forming a complex with DNA repair protein XPA, RASSF1A enhances XPA’s full repair activity and sustains genomic fidelity during replication stalling^64^. In addition, the RASSF1A-LATS signaling axis restricts CDK2-mediated phosphorylation of BRCA2 and thereby supports BRCA2’s function and thus stabilization of the replication fork^53^.

Overall, the occurrence of cytokinesis defects after RASSF1A depletion possibly acts as the initial trigger for cell transformation, promoting tetra- and aneuploidy as well as genomic instability, which are early hallmarks of almost all types of cancer^108^. Accordingly, the tumor-suppressor activity of RASSF1A has been reported to reside mainly in its ability to restrict cell cycle progression^35^.

### RASSF1A and cell death pathways

Cues that drive cell growth and division also induce cell death. In an abnormal cell proliferation scenario, such as cancer, cells adopt a variety of strategies to overcome the pathways which are implicated in cell death^109^. RASSF1A regulates several critical signaling pathways involved in the control of programmed cell death.

#### RASSF1A and apoptosis

**Fig. 5:**
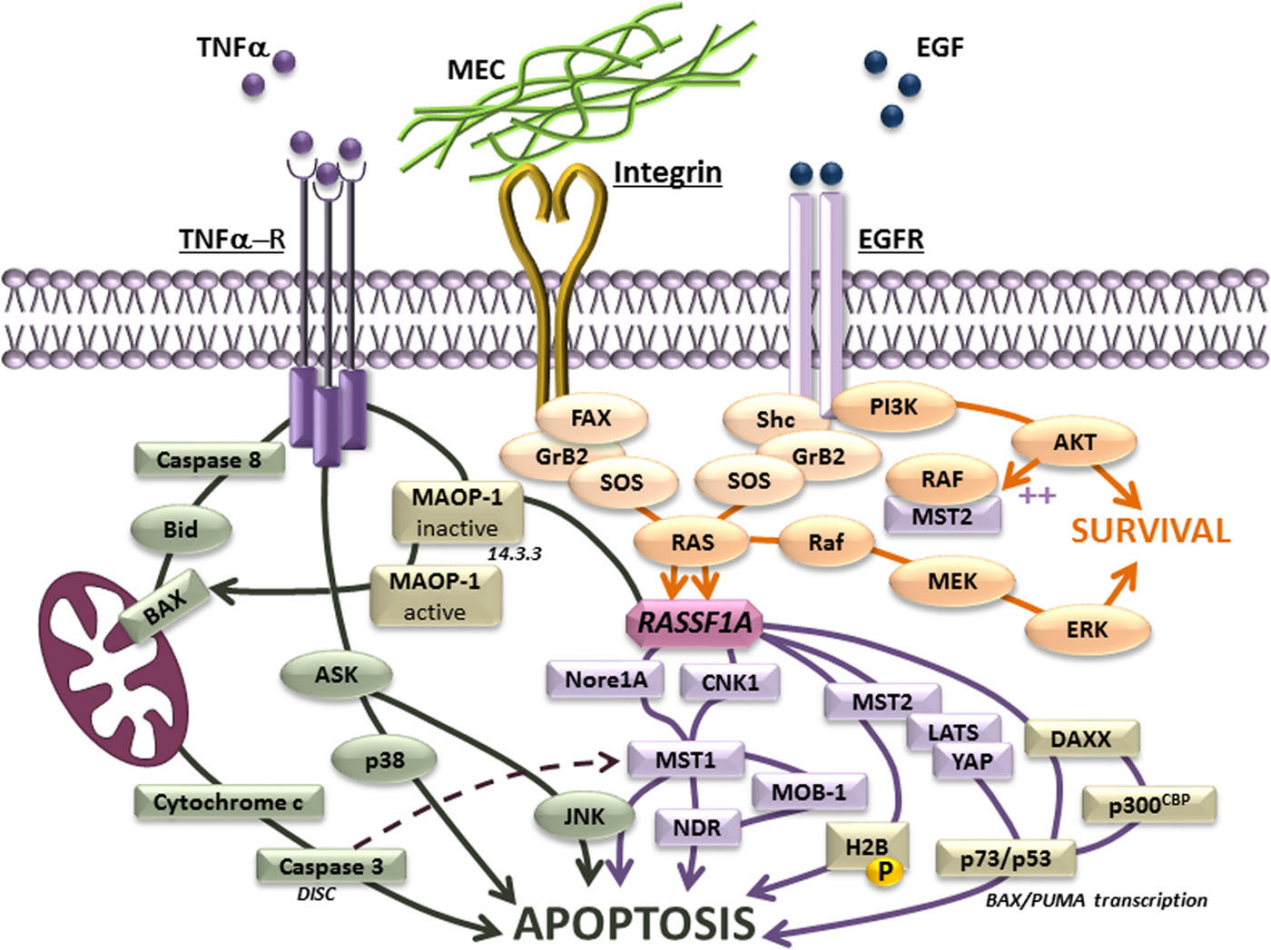
RASSF1A as a pro-apoptotic protein. In contrast to proto-oncogenic activity of Ras to enhance cell survival, RASSF1A positively controls apoptosis through several pro-apoptotic pathways^60,75,111^.

Apoptosis, the best-characterized form of programmed cell death^110^, is positively regulated by RASSF1A through several pathways^60,75,111^ (Fig. 5). RASSF1A’s pro-apoptotic action is mediated through direct interaction with the Hippo kinases, MST1 and MST2, preventing their dephosphorylation^112^ and inactivation^113^. In the basal condition, RASSF1A keeps MST1 inactive^114^, whereas the stimulation with death receptors leads to the activation of MST1 through auto-phosphorylation and the association of the scaffold protein CNK1 to the RASSF1A/MST1 complex, which leads to Caspase-3 activation and apoptosis^115,116^. Activated caspase-3 removes the auto-inhibitory domain of MST1, and increases its activity (~10-fold)^115,117^. The liberated MST1’s fragment translocates to the nucleus, activates the Jun N-terminal kinase (JNK) and phosphorylates the Ser-14 from Histone 2B, which results in chromosome condensation and DNA fragmentation, the most prominent morphological manifestations of apoptosis^112^. Through another signaling pathway, RASSF1A removes MST2 sequestration with the RAF-1 protein, enhancing its activated auto-phosphorylated form^66^. Subsequently, MST2 activates the NDR kinases, which in turn phosphorylate the YAP1 transcriptional co-activator. Conversely, on its unphosphorylated state, YAP translocates into the nucleus where it forms a complex with p73/p53, promoting the transcription of pro-apoptotic genes such as Bax and PUMA to initiate apoptosis^10,66^. Thereby, endogenous RASSF1A controls apoptosis via inhibitory action on YAP. In the context of RASSF1A pro-apoptotic action, the role of other YAP targets implicated in the control of cell death, such as inhibitor of apoptosis protein family (IAPs)^118^, requires further investigation.

RASSF1A is also directly involved in the apoptosis extrinsic pathway, activated by extracellular signals through death receptor family proteins, including FAS (Fas cell surface death receptor), TNF (tumor necrosis factor), and TRAIL (tumor necrosis factor ligand superfamily). Following their stimulation, RASSF1A is no longer protected by the 14–3–3 scaffold protein and forms a new complex with MOAP-1 (modulator of apoptosis-1) which is recruited by death receptors^28,61^. RASSF1A/MOAP-1 association then promotes Bax conformational change, the release of mitochondrial cytochrome-c and activation of caspases^16^. Conversely, the absence of RASSF1A prevents activation of the Bax by death receptors, confirming RASSF1A’s central role in the modulation of apoptosis’s extrinsic pathway^60^.

RASSF1A also contributes to apoptosis independently of caspases. In response to the DNA damage and the phosphorylation of its serine 131 by the ATM kinase^18^, RASSF1A interacts with MDM2 (murine double minute 2). MDM2 is an E3 ligase capable of binding to the N-terminal portion of p53, thereby inhibiting its transcriptional activity, and inducing p53 degradation by the proteasome after ubiquitinylation. The association of RASSF1A with MDM2 after DNA damage, disrupts MDM2’s interaction with DAXX and HAUSP proteins, leading to MDM2 ubiquitination, thus promoting the stabilization and activation of p53^119,120^. Accordingly, RASSF1A appears to play an important role in the pro-apoptotic function of p53, independently of caspases. Overall, RASSF1A influences the function of both p53 and p73 to maintain genomic stability^4,121^. In fact, as previously described, the mutation of the RASSF1A’s ATM phosphorylation site has already been reported in some cancers^19^.

#### RASSF1A and autophagy

Autophagy is a highly regulated catabolic process, involving the formation of a double-membrane cisterna (autophagosomes), in which protein aggregates, damaged organelles, cellular debris, and pathogens are trapped for degradation and/or recycling^122^. Death occurs as the cell digests its own proteins and organelles beyond an irreversible point^123^. Since MTs are involved in biogenesis, transport and fusion of mature autophagosomes with lysosomes^124,125^, RASSF1A’s implication in autophagy seems critical^126,127^. Consistently, RASSF1A binds directly to MAP1S^43^, the bridge connecting autophagy with MTs and mitochondria, affecting autophagosomal biogenesis and degradation^10^. RASSF1A also promotes autophagy maturation by recruiting autophagosomes on RASSF1A-stabilized acetylated MTs through MAP1S^54,127,128^.

Furthermore, numerous studies provide an important link between the members of the Hippo pathway and autophagy regulation^129,130^. Consequently, the association of RASSF1A with these members adds a further layer of complexity to the regulation of autophagy by RASSF1A. In agreement, RASSF1A, through its interaction with MST1, enhances autophagy initiation *via* suppressing the PI3K-AKT-mTOR pathway^54^, one of the principal pathways implicated in suppressing autophagy initiation^131^. Altogether, RASSF1A enhances autophagy initiation and maturation to activate autophagy flux.

#### Putative RASSF1A role on ferroptosis via YAP

Ferroptosis, a cell death process driven by iron-dependent lipid peroxidation, is promoted by YAP activation^132^. By inducing YAP activation, RASSF1A depletion^56^, could thus play a role in ferroptosis. Consistently, cancer cells with RASSF1A/NF2/Hippo alterations, such as mesothelioma cell lines, are sensitive to ferroptosis-inducing drugs^132^.

### RASSF1A and inflammation

Chronic Inflammation has long been implicated as an essential factor in carcinogenesis^133,134^, and it is believed that some mediators of inflammation could play a critical role in carcinogenesis. For instance, such mediators could induce persistent epigenetic changes in the primary tumor cells that affect fundamental processes necessary for generating cell variants with metastatic ability^135^. It has been shown for example that interleukin (IL)-6, an inflammatory cytokine crucial in the host immune defense response, increases DNA methyltransferase-1 (DNMT-1) that play a key role in the maintenance of DNA methylation^136^. Interestingly, RASSF1A expression is significantly downregulated in IL-6 overexpressing cells through activation of DNMT1 and higher percentage of CpG methylation^137,138^. Moreover, RASSF1A is also implicated in the protection pathways against inflammation, as RASSF1A-deficient tumors presented a marked increase in inflammation and IL-6 production, in addition to the abundant presence of macrophage marker positive cells^69^. Consistently, RASSF1A-knockout mice displayed clinical symptoms of inflammatory disease^139^. Furthermore, RASSF1A-deficient transgenic mice, as well as RASSF1A-deficient tumors showed an elevated level of IL-6 production^69,139^. It is of note here that YAP nuclear accumulation, observed in RASSF1A-depleted cells^56^, also increases the transcription of IL-6 gene^140^. Considering the role of IL-6 in tumor initiation or metastasis as inducing EMT^141,142^, we can argue a collaborative relationship between RASSF1A depletion, YAP nuclear accumulation, and elevated IL-6 in carcinogenesis.

Accordingly, another argument considering the potential cooperation of RASSF1A and inflammation comes from recent data, which have shown the negative control of RASSF1A on the NFκ-B pathway^143,144^. NF-κB, a transcription factor, is introduced as central to inflammation-induced tumor progression and malignant transformation^83,145^. Upregulation of NF-κB promotes invasiveness, metastasis, proliferation, and anti-apoptosis of cancer cells^146,147^. Thus, RASSF1A tumor inactivation may also play a central role in inflammation-regulated progression of cancer.

## Conclusions and perspectives

**Fig. 6:**
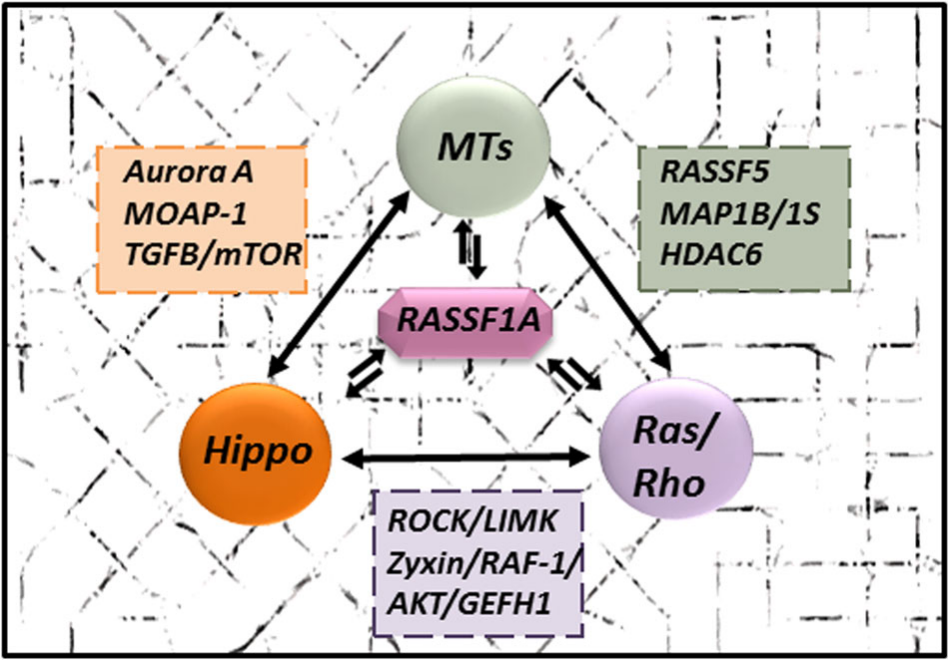
**As a tumor-suppressor gene, RASSF1A mainly acts as a crossroad of three intertwined molecular signaling mechanisms including Ras/Rho GTPases, MTs, and Hippo pathway**.

Over the last 20 years, research on RASSF1A has uncovered a wide spectrum of functions for RASSF1A as a tumor and metastasis suppressor protein, as a nexus for the coordination of numerous signaling pathways (Fig. 6). RASSF1A silencing is related to deregulation of cell proliferation, cell death, invasion, and to distant metastasis. Here, we have presented a general overview of these aspects of RASSF1A biology and of the vast networks through which RASSF1A acts. However, capturing the whole complexity of the RASSF1A function is beyond the scope of this article. The newly discovered implication of RASSF1A in the interaction with the hypoxia inducible factor-1α (HIF-1 α), which enhances the activation of the glycolytic switch, hints at the complexity of RASSF1A’s activity^148^. It is important to bear in mind that RASSF1A act through not only heterodimers interactio, but potentially even hetero-trimers formation with an extensive array of other regulatory proteins involved in cellular signal transduction of proliferative and anti-proliferative pathways. Thus, an important focus of future studies must be the identification of the mechanism(s) by which the cell orchestrates these interactions in a spatio-temporal and context-specific manner. Given the extensive synergy between the RASSF1 family members, another important issue to investigate is the influence of other RASSF1 members in promoting tumor progression. Future work is required to untangle the complexity of RASSF1A’s scaffold activity and the role that other signaling pathways such as Ras GTPases, or Hippo play in its function.
